# Epigenetic programs shaping lung metastasis in triple-negative breast cancer

**DOI:** 10.21203/rs.3.rs-7659153/v1

**Published:** 2025-11-05

**Authors:** Diego Marzese, Andres Bedoya-Lopez, Javier Orozco, Miquel Ensenyat-Mendez, Betsy Valdez, Sandra Iñiguez-Muñoz, Alexander Boiko, Huiwen Xie, Maggie DiNome, Pere Llinàs-Arias

**Affiliations:** IdISBa

**Keywords:** Metastasis, triple-negative breast cancer, DNA methylation, lung metastasis

## Abstract

Triple-negative breast cancer (TNBC) is a major cause of cancer mortality, with distant metastases presenting a significant clinical challenge. While epigenetic mechanisms like DNA methylation are known to influence TNBC progression, their specific role in driving metastasis remains underexplored. To address this gap, we performed comprehensive multi-omics analysis, integrating epigenomic and transcriptomic profiles from TNBC xenograft models and patient cohorts. Genome-wide DNA methylation profiling of primary tumors, lymph nodes, and lung metastases from xenografts revealed pronounced global hypomethylation in lung lesions, consistent with findings from the clinical cohort. Promoter-methylation changes were enriched in pathways linked to invasion and proliferation, and transcriptomic integration identified 22 epigenetically regulated genes. Among these, elevated *AK1*, *SLC2A5*, *TPI1*, and *ZBTB17* expression correlated with a higher risk of lung dissemination. These findings highlight altered DNA methylation as a driver of TNBC lung colonization and identify candidate prognostic markers, emphasizing the importance of epigenetic reprogramming in organ-specific lung metastases.

## INTRODUCTION

Breast cancer (BC) is the most commonly diagnosed tumor and the leading cause of cancer-related mortality among women^1^, with distant metastases accounting for approximately 90% of these deaths^2^. Patients suffering from triple-negative breast cancer (TNBC), defined as BC with the absence of hormone receptors and lack of HER2 amplification, have an increased likelihood of developing metastases^3^. This invasion–metastasis cascade is a complex, multi-step process that begins with local invasion and progresses until a few disseminated cancer cells succeed in completing the final step of colonization to distant organs^4^.

The lung, bone, brain, and liver represent the most frequent sites of distant metastases in BC, with TNBC exhibiting a particularly high propensity to metastasize to the lungs^2^. Notably, lung metastases occur in approximately 40% of TNBC tumors, compared to only 20% in non-TNBC^2,5^, and the prognosis in such cases is poor, with a median survival of only 11 months following treatment^6^. This unfortunate outcome is principally attributed to the limited number of treatment options available for inoperable lesions^7^.

Identifying the alterations and timing that endow tumor cells their metastatic capabilities is essential for the development of effective therapeutic strategies. Despite ongoing efforts, the mechanisms remain incompletely understood and subject to debate. Emerging evidence describes epigenetic mechanisms^8^, such as DNA methylation (DNAm), as central regulators in the initiation, progression, and dissemination of BC. For example, hypermethylation of metastasis suppressor genes such as *BRMS1* has been associated with lymph node metastases in TNBC patients^9^; epigenetic silencing of *CDH1* has also been involved in BC metastases^10^. Additionally, previous studies have shown that DNA hypermethylation of leukocyte-related genes can serve as an immune-evasive mechanism in TNBC lesions^11,12^.

However, the entire landscape of specific TNBC-epigenetic mechanisms driving progression from primary tumors to both local and distant sites remains poorly characterized. In this study, we profiled genome-wide DNAm across more than 860,000 CpG sites in TNBC xenograft mouse tumors (n = 11) exhibiting both regional (lymph node) and distant (lung) metastases. We then integrated these data with patient-derived multi-omic datasets from the AURORA US Metastasis Project (n = 153; 51 primary tumors and 102 metastases) and other independent clinical cohorts to comprehensively interrogate epigenetic programs underlying TNBC dissemination. Our analyses revealed an aberrantly methylated landscape in TNBC lung metastases, highlighting specific epigenetically regulated genes with potential to enhance metastatic capacity at this clinically challenging site, where therapeutic options remain limited. Together, these findings provide a framework for elucidating TNBC-specific epigenetic mechanisms driving progression from primary tumors to both regional and distant metastatic sites.

## RESULTS

### TNBC lung metastases have a distinct epigenetic landscape compared with paired primary tumors and lymph node metastases

**Figure 1:**
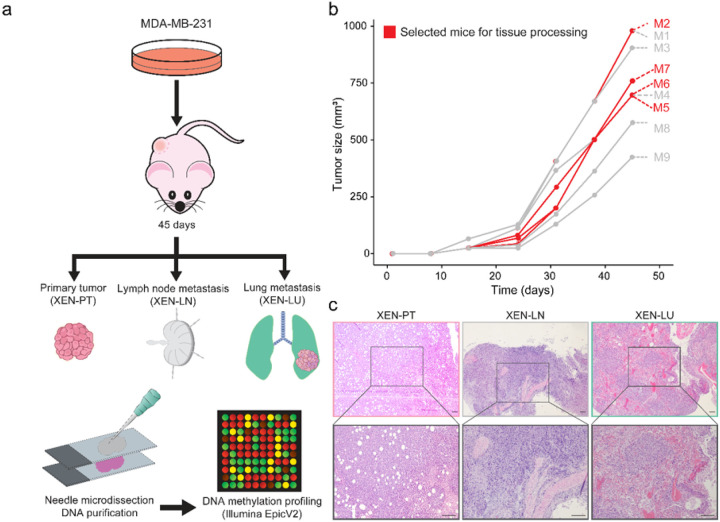
Generation of TNBC cell line-derived xenografts and tissue processing for epigenomic profiling. **a)** Schematic representation of the subcutaneous xenograft model used to study TNBC metastases in nude mice. **b)** Tumor growth curve over 45 days following subcutaneous injection. Red lines note those animals selected for tissue processing. **c)**Representative H&E staining of primary tumors (XEN-PT), lymph node metastases (XEN-LN), and lung metastases (XEN-LU) derived from TNBC xenografts. Upper panels: 4× magnification; lower panels: 10× magnification (scale bar = 150 μm).

To study epigenetic adaptation of TNBC lung metastases *in vivo*, we established mammary fat pad tumors in mice by subcutaneous injection of the well-characterized MDA-MB-231 cell line^13^. These xenografts, referred to as XEN-PT for primary tumors, were maintained for 45 days before euthanasia. Upon dissection, metastatic lesions were confirmed in lymph nodes (XEN-LN) and lungs (XEN-LU, Fig. 1a–b). Representative hematoxylin and eosin (H&E) staining confirmed the histological features of primary tumors and metastatic sites (Fig. 1c).

**Figure 2:**
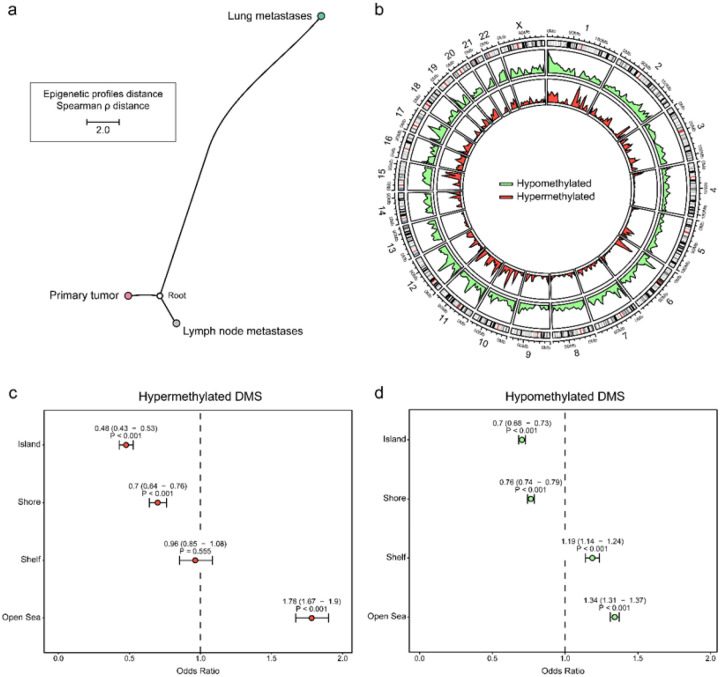
DNA methylation differences between TNBC lung and lymph node metastases and primary tumors from our xenograft murine models. **a)** Phylogenetic tree representing DNAm-based Spearman ρ distance between tumor samples from different sites. **b)** Circus plot displaying all the DMS across the genome between lung metastases, compared to lymph nodes and primary tumors. **c)** Forest plot displaying the odds ratio with 95% confidence intervals for hypermethylated and **d)** hypomethylated DMS across different CpG genomic regions.

These lesions were subsequently processed for epigenomic profiling. By comparing DNAm levels of the top 5,000 most variable genomic regions, we found that lung metastases displayed a distinct epigenetic profile compared with lymph node metastases and matched primary tumors (**Supplementary Fig. 1a**). The same pattern was maintained when we examined the top 10,000 and even 25,000 most variable regions (**Supplementary Fig. 1b-c**). The distinction was further confirmed by phylogenetic distance analysis, where TNBC lung metastases showed greater separation from the other groups (Fig. 2a), indicating a distinct epigenetic landscape.

Comparing lymph node metastases and primary tumor specimens revealed only 1,302 differentially methylated sites (DMS), including 579 hypermethylated and 723 hypomethylated CpG sites (**Supplementary Fig. 1d**). In contrast, we identified significantly higher DMS when comparing lung metastases with both primary tumors and lymph node metastases (31,269 and 31,159, respectively). Among these, 3,089 and 5,236 sites were hypermethylated, while 28,180 and 25,923 were hypomethylated (**Supplementary Fig. 1d**), indicating a clear trend towards a loss of DNAm marks in distant metastases compared to regional metastases or primary tumors. These results suggest a lung-specific epigenetic reprogramming, potentially reflecting adaptation to the pulmonary microenvironment.

### Epigenome adaptation shifts towards a widespread hypomethylation during TNBC lung metastases

Building on our previous findings, we focused on dissecting the specific differences of lung metastases. Due to the epigenetic similarities between primary tumor specimens and regional metastases and the relatively low number of cases per group, we compared lung metastases with the combined lymph node and primary carcinomas. A total of 40,204 DMS were identified in this comparison. Among them, 35,580 CpG sites were hypomethylated and 4,624 were hypermethylated, indicating a tendency toward global hypomethylation in lung metastases (Fig. 2b). Specifically, DMS were significantly enriched on several chromosomes (adjusted P < 0.05), including chromosomes 7, 13, and X, whereas chromosomes 16, 19, and 20 showed relative depletion (**Supplementary Fig. 2a**).

Interestingly, hypermethylated CpG sites were most enriched in open sea regions and less common in CpG islands and shores, while hypomethylated CpG sites showed a similar but less pronounced distribution (Fig. 2c–d). To further investigate these methylation changes, we assessed whether they were enriched in specific chromatin states, given the activation of multiple cascades during metastasis. These analyses showed that hypermethylation in lung metastases was mainly enriched in heterochromatin, regions typically linked to transcriptional repression and markedly reduced in areas of active transcription. Hypomethylated sites displayed a comparable distribution, although the differences were less pronounced (**Supplementary Fig. 2b–c**). Together, these results point to broad epigenetic shifts from primary tumors to distant metastases, affecting both heterochromatin and transcriptionally active regions. This pattern, combined with the global hypomethylation observed in lung metastases, suggests an epigenetic loosening that may enable the activation of invasive and adaptive transcriptional programs.

### Epigenetic rewiring of promoter methylation involves genes linked to metastatic processes

It is well established that methylation of the gene promoter influences gene expression. Typically, hypermethylation of the promoter region leads to gene inactivation, while hypomethylation tends to result in increased gene expression^14^. To determine whether there were gene networks primarily dysregulated and mediated by these epigenetic mechanisms during lung metastases, we focused on protein-coding genes with at least two DMS within their promoter regions. We identified 1,373 genes exhibiting such epigenetic differences, with the majority being hypomethylated (n = 1,176), while a smaller subset was either hypermethylated (n = 48) or displayed mixed methylation patterns (n = 149) at their promoters.

**Figure 3:**
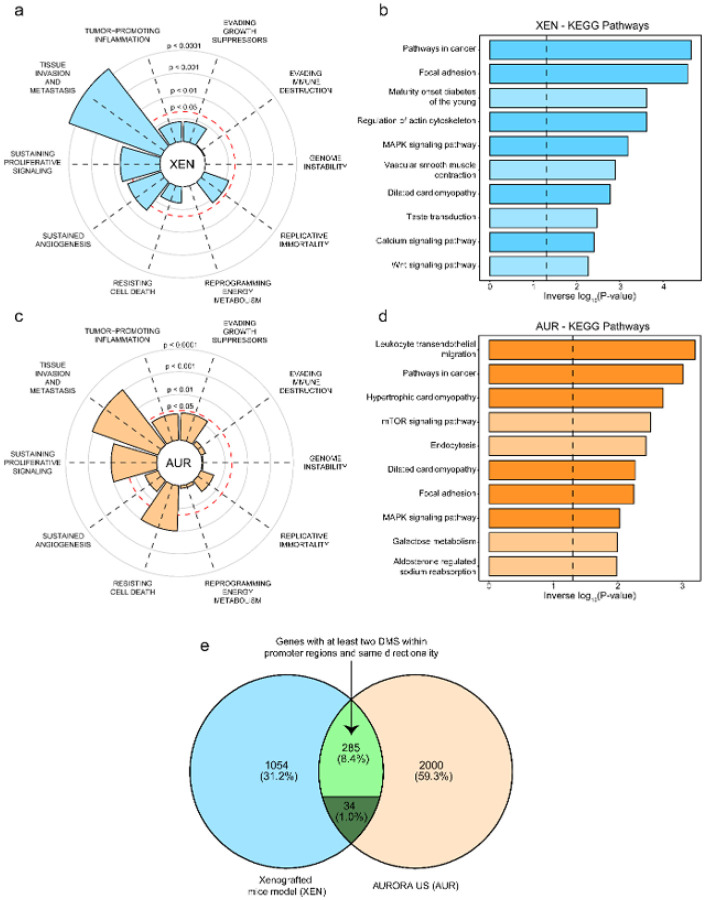
Epigenomes from our murine model and the AURORA US cohort converge in dysregulated DNA methylation patterns across cancer-related networks and genes. **a)** Cancer hallmark enrichment analysis based on protein-coding genes harboring at least two DMS within their promoter regions in our XEN model. **b)** Top 10 enriched KEGG pathways using the same gene set from the XEN model. The x-axis shows the inverse log_10_ of the P-values. Darker bars represent pathways also significantly enriched in the AUR cohort. **c)** Cancer hallmark enrichment analysis using protein-coding genes with at least two DMS within their promoter regions in the AUR cohort. **d)** Top 10 enriched KEGG pathways identified using the same criteria. The x-axis shows the inverse log_10_ of the P-values. Darker bars represent pathways also significantly enriched in the XEN model. **e)** Venn diagram showing the overlap of genes with promoter-level methylation changes. Light green shows the genes with consistent differential methylation in both cohorts, between the XEN and AUR cohorts, while dark green shows those genes with discordant differential methylation.

We then performed functional enrichment analysis on these genes. Using the Cancer Hallmark gene set, which encompasses multiple cancer-related networks, we found that most of these genes, potentially regulated by methylation changes, were significantly enriched in pathways associated with tissue invasion and metastasis, sustained proliferative signaling, and angiogenesis (Fig. 3a).

Interestingly, KEGG analysis revealed that well-established metastasis drivers were primarily dysregulated in this process, including the Wnt/β-catenin and MAPK signaling pathways (Fig. 3b). These findings suggest that epigenetic alterations may play a key role in driving metastatic progression to the lungs by influencing critical oncogenic processes, reflecting a broader epigenetic shift that imparts metastatic properties to tumor cells.

### Patient-derived epigenetic profiling supports the impact of hypomethylation in TNBC lung metastases

We validated our findings by using data from clinical patients enrolled in the AURORA US cohort^11^. This dataset included paired DNAm and transcriptomic profiling of 19 TNBC samples, including six treatment-naïve primary tumors (AUR-PT), four lung metastases (AUR-LU), and nine lymph node metastases (AUR-LN) (**Supplementary Table 1**).

Interestingly, based on a phylogenetic tree constructed using the 5,000 most variable sites, patient-derived lymph node metastases exhibited a methylation profile more closely related to lung metastases than to primary tumors. This pattern was not observed in our mouse model (**Supplementary Fig. 3a**, Fig. 2a). When we compared methylomes, we identified only 4,030 hypomethylated and 238 hypermethylated CpG sites between lung and lymph node metastases (**Supplementary Fig. 3b**). We conducted separate analyses comparing lung metastases with primary tumors and lymph node metastases with primary tumors. Both comparisons revealed a trend toward hypomethylation, supporting the notion of widespread DNA hypomethylation during metastatic progression. In particular, lung metastases exhibited 35,899 hypomethylated and 5,566 hypermethylated CpG sites relative to primary tumors, whereas lymph node metastases showed a less pronounced shift, with 19,197 hypomethylated and 8,467 hypermethylated sites (**Supplementary Fig. 3b**).

The similarities observed in our murine model and the patient cohort indicate that global hypomethylation may be a hallmark during the transition from primary TNBC tumors to metastases, with distant lesions exhibiting a more pronounced methylation loss than regional ones. The closer similarity between lung and lymph node metastases in patients, compared to mice, may reflect differences in metastatic progression timelines or microenvironmental influences.

### Shared epigenetic alterations in TNBC lung metastases across xenograft and patient-derived data display tissue invasion and metastasis phenotypes

Given that the most pronounced epigenetic differences were observed in distant metastases, we focused our analysis on lung metastases relative to primary tumors. Building on our previous pipeline, we specifically analyzed protein-coding genes harboring at least two DMS within their promoter regions. Our epigenomic analysis revealed that 2,319 genes satisfied this criterion. Similar to our model, most genes were found hypomethylated (n = 1,908), while a smaller number were either hypermethylated (n = 135) or exhibited mixed methylation patterns (n = 276).

Additionally, we found that genes potentially regulated by DNAm in patient samples were enriched in pathways associated with sustained proliferative signaling, tissue invasion and metastasis, and resistance to cell death (Fig. 3c). Interestingly, the latter was not enriched in our model, underscoring the hostile environment that cells encounter during colonization, a process that is more critical in the human host than in immunodeficient mice. We next investigated whether these genes harboring DMS in promoter regions were enriched in specific KEGG pathways. Again, several pathways related to oncogenic processes were enriched, with most of them also enriched significantly in our models (Fig. 3d), including MAPK signaling, focal adhesion, and other pathways in cancer.

Although multiple routes can ultimately reach the same biological outcome, we sought to examine which genes were commonly affected by these methylation marks in both our XEN model and the AURORA US cohort. Notably, similar patterns were observed, with 284 genes exhibiting concordant methylation across both datasets, while a minority of 34 genes displayed discordant patterns and were therefore removed from downstream analyses (Fig. 3e). This shared 284-gene methylation signature represents an epigenetic program that appears to be associated with lung tropism.

### Epigenetic mechanisms shaping transcriptomic programs in TNBC lung metastases

**Figure 4:**
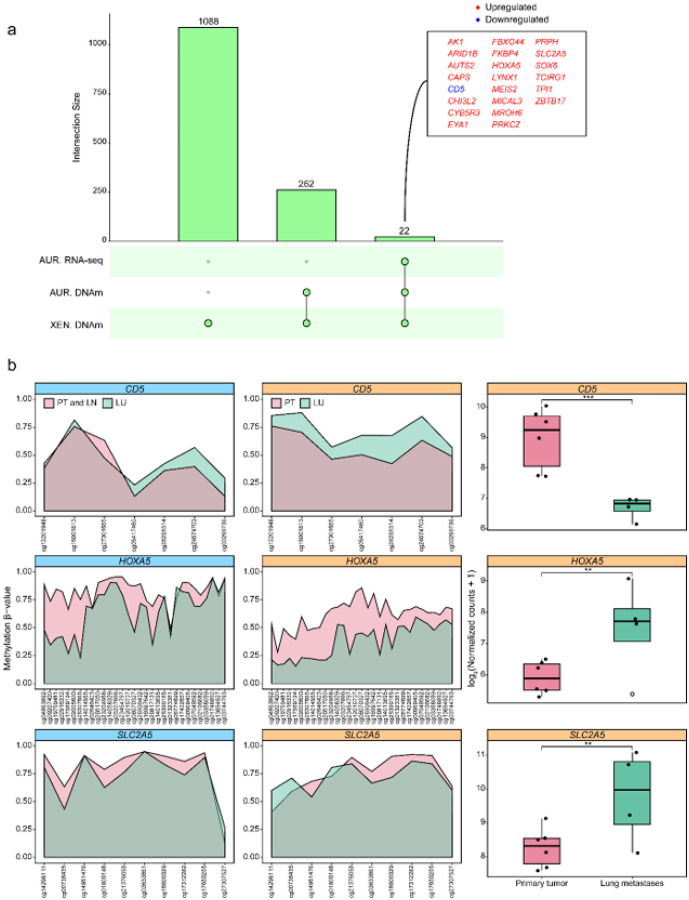
Promoter-level epigenetic rewiring impacts gene expression in TNBC lung metastases. **a)** Upset plot showing genes with promoter-level differential methylation that are unique to or shared between the XEN model (n = 1,088) and the AURORA US cohort (n = 262), and that also display significant and consistent changes in mRNA expression in the AURORA US cohort (n = 22). A total of 22 genes were identified, 21 upregulated (red) and 1 downregulated (blue). **b)** Promoter methylation levels in the XEN model (cyan) compared with methylation and gene expression levels in the AUR cohort (orange) for *CD5*, *HOXA5*, and *SLC2A5*. Methylation is shown as area plots (in green LU) overlaid on pink regions representing PT or combined PT and LN samples, distributed by CpG position and its probe ID. Gene expression is shown as boxplots. Statistical significance was assessed using the Wald test from the DESeq2 pipeline: ** *P-value* < 0.01; *** **P-value**< 0.001.

To assess whether these epigenetic alterations directly impact gene expression, we analyzed the transcriptomic profiles of the same tumors. This analysis revealed 1,146 downregulated and 1,220 upregulated genes in lung metastases relative to primary tumors, revealing a core set of transcriptional changes associated with lung metastatic onset and progression (**Supplementary Fig. 3c**). Notably, 22 genes appeared to be epigenetically regulated, displaying DNAm changes in our XEN model alongside consistent alterations in both DNAm and gene expression in the AURORA US dataset (Fig. 4a). For instance, *CD5*, *HOXA5*, and *SLC2A5* exemplified distinct patterns of promoter methylation; while *CD5* exhibited extensive promoter hypermethylation associated with reduced gene expression, *HOXA5* and *SLC2A5* showed hypomethylation, widespread in *HOXA5* and focal in *SLC2A5*, both associated with increased transcript levels (Fig. 4b).

To determine if these genes were specifically dysregulated during lung metastasis or if they represented a common biological barrier during the colonization phase in other organs, we analyzed their expression profiles. Interestingly, several genes, such as *ARID1B, SLC2A5*, or *PRPH*, seemed to be lung-specific, while others, like *PKRCZ* or *TPI1*, showed a broader pattern with no distinction of the metastatic niche (**Supplementary Fig. 4**). As in the epigenome, local metastases to lymph nodes showed less pronounced differences than distant metastases.

The identification of genes with lung-specific epigenetic and transcriptional changes highlights candidate effectors of organ-specific metastasis. These findings suggest that epigenetic alterations in a subset of genes directly influence their transcriptional regulation, defining a core set of genes dysregulated in TNBC metastases, with several directly related to specific lung tropism.

### A core gene set of epigenetically regulated genes may play a role in the lung tropism of TNBC tumors

**Figure 5:**
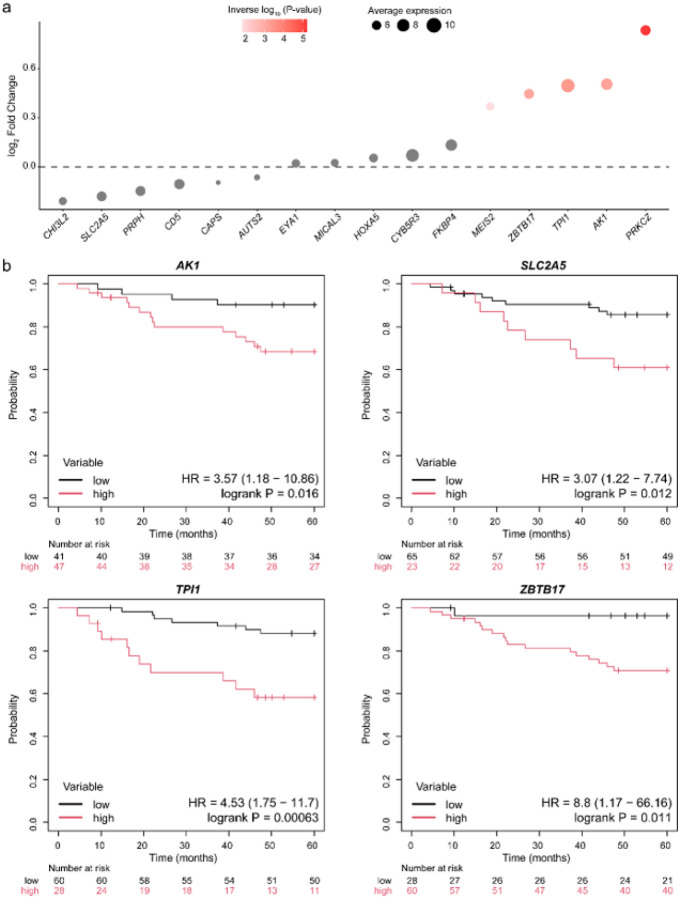
Association of dysregulated genes between lung TNBC metastases and primary tumors with lung metastasis-free survival for TNBC primary tumors. **a)** Cascade plot showing gene expression comparison between xenograft-derived cell lines with lung metastatic potential and the parental cell line. **b)** LMFS rates for TNBC patients stratified based on the expression of four genes (*AK1*, *SLC2A5*, *TPI1*, and *ZBTB17*). The Kaplan–Meier curves were obtained using the Kaplan–Meier Plotter web tool and include HR and 95% CI estimated from the associated unadjusted Cox proportional hazards model and the log-rank test P-value.

To further evaluate the functional relevance of these genes, we examined gene expression data from an immunodeficient mouse model injected with MDA-MB-231 cells that metastasized to the lung, compared to the parental cell line^15^. Out of the original subset of 22 genes, only 16 were available for this study (lacking gene expression from *ARID1B*, *FBXO44*, *LYNX1*, *MROH6*, *SOX6*, and *TCIRG1*). Among these, five genes remained consistently upregulated (*MEIS2*, *ZBTB17*, *TPI1*, *AK1*, *PRKCZ*; Fig. 5a), underscoring their importance during the step from primary tumors to lung colonization in an independent xenograft model.

Given the preferential spread of TNBC cells to the lung, we hypothesized that some of these alterations may not merely reflect metastatic adaptation but could actively contribute to a lung-tropic phenotype when already present in the primary tumor. To investigate this, we analyzed an independent TNBC cohort (n = 88) of primary tumors with annotated lung metastasis-free survival (LMFS). Notably, six of the 16 genes were significantly linked to an increased risk of lung dissemination when dysregulated in the primary tumor, while others were nearly significant, highlighting their potential clinical relevance (**Supplementary Fig. 5**). Among these, four genes displayed consistent directionality in their differences between metastases and primary tumors. Specifically, higher expressions of *AK1*, *SLC2A5*, *TPI1*, and *ZBTB17* in primary TNBC tumors were significantly linked to increased risk of lung metastases (Fig. 5b). In contrast, low expression of *AUTS2* and *FKBP4*, both upregulated in lung metastases from the AURORA US cohort, was associated with worse LMFS, suggesting a gene expression shift after the step of lung colonization (**Supplementary Fig. 5**).

Together, these findings reinforce the idea that several genes controlled by epigenetic mechanisms are key contributors to lung-specific metastasis in TNBC, meriting further investigation as potential prognostic markers or therapeutic targets.

## DISCUSSION

In this exploratory study, we provide evidence that epigenetic mechanisms may play an important role during the progression of TNBC from primary sites to distant metastases, specifically during lung colonization, which represents a critical challenge in the current clinical settings.

Interestingly, we found that DNAm is broadly lost during the metastatic cascade, with lung metastases displaying a more pronounced shift compared to lymph node metastases. Such global hypomethylation has long been recognized as an early epigenetic abnormality in human tumors^16^, with previous studies linking it to the activation of genes involved in tumor invasion and metastasis^17^. This becomes particularly relevant given that, although more than 10,000 cancer genomes have been sequenced, clear evidence of specific “driver mutations” promoting metastasis remains elusive^10,18^. These observations, along with our results, highlight the need to explore alternative mechanisms, including epigenetic reprogramming, as the basis of metastatic progression.

Our study revealed a coordinated epigenetic remodeling process that affects the promoter regions of multiple genes. These genes reflect broader dysregulation across several cancer hallmarks, with tissue invasion and metastatic progression being the most highly affected processes. Most of these genes belong to well-characterized cancer-related networks, including MAPK and Wnt signaling pathways^19,20^. This is consistent with evidence from other xenograft models showing that Wnt signaling can couple cancer cell self-renewal with the expression of EMT transcription factors, ultimately promoting tumor seeding and lung metastasis in TNBC cells^21^. Other processes were not epigenetically enriched in our XEN models but were altered in the AURORA US patient cohort. One possible explanation is that xenografts are performed in immunocompromised mice, where metastatic cells bypass selective pressures imposed by the immune system. In patients, by contrast, TNBC cells must evolve strategies to evade or suppress immune surveillance, which may lead to convergent epigenetic programs across lymph node and distant metastases. This highlights the role of the immune context in shaping organ-specific metastatic trajectories and may account for differences observed between mouse and human data. This limitation is not unique to our study but rather inherent to most *in vivo* experimental models on immunocompromised mice.

To identify potential theragnostic biomarkers and key regulators of the breast-to-lung metastatic transition, we integrated DNAm with gene expression data. Typically, promoter hypermethylation leads to gene silencing, whereas hypomethylation is associated with increased gene expression^14^. We identified a panel of 22 epigenetically regulated genes. Among these, *SLC2A5*, *SOX6*, and *PRPH* were specifically overexpressed in lung metastases, while *PRKCZ* and *TPI1* were broadly upregulated across multiple metastatic sites. This pattern suggests that some genes may exert niche-specific functions that facilitate the colonization of particular organs, while others support metastatic outgrowth regardless of the target site. For instance, *SOX6*, a lung-specific dysregulated gene, has been implicated in lung embryonic development, potentially reflecting an adaptive advantage to this tumor–niche crosstalk^22^.

The tendency of TNBC cells to metastasize to the lung suggests that some of the observed molecular alterations may actively promote a lung-tropic phenotype rather than merely reflecting adaptations after dissemination. In particular, four genes, *AK1*, *SLC2A5*, *TPI1*, and *ZBTB17*, showed consistent upregulation from primary tumors to metastases, and their higher expression in primary TNBC tumors was associated with an increased likelihood of LMFS. These findings support the idea that primary TNBC tumors may already harbor subpopulations of cells with an inherent predisposition to colonize the lung. Consistent with this, Xiao et al. reported that tumor-secreted cathepsin C promotes TNBC lung metastasis by modulating neutrophil infiltration and inducing neutrophil extracellular trap formation^23^, highlighting the role of proteins expressed in the primary tumor in conferring a lung-tropic phenotype.

Whether such subpopulations evolve in parallel to target lymph nodes and lungs independently, or whether lymph node colonization represents an intermediate step toward lung dissemination, remains an open question in TNBC. Future studies leveraging DNAm–based phylogenetic reconstruction or single-cell multi-omics may help disentangle these evolutionary trajectories and identify early lung-tropic subclones.

While our study provides valuable insights, it also has limitations. The lack of transcriptomic data from our XEN model diminishes our understanding of the epigenetic impact on gene expression. However, this gap is partially addressed by validation in the AURORA US cohort, which includes matched epigenomic and transcriptomic data from clinical tumor samples, as well as transcriptomic profiling from independent TNBC cell line-derived xenograft models. Additionally, our analysis was limited to protein-coding genes, excluding other potential regulatory mechanisms that DNAm marks may influence, such as long non-coding RNAs and antisense transcripts. Another limitation of our study is the relatively small sample size in the epigenetic profiling cohorts, including both our XEN model and the AURORA US dataset, which may reduce the statistical power of our analysis. Finally, our analysis was restricted to promoter regions, despite methylation in gene bodies or enhancers also playing roles in gene regulation and cancer progression^24,25^. Despite these limitations, our integrated multi-omics approach across independent cohorts and models provides a robust foundation for understanding the epigenetic mechanisms driving TNBC lung metastases and highlights avenues for future research to fully elucidate this metastatic epigenetic landscape.

## METHODS

### Ethical approval

All mouse experiments were approved by and performed according to the guidelines of the Institutional Animal Care and Use Committee of the United States (IACUC, code #AUP-19–04). All publicly available datasets used in this study were obtained from established repositories with appropriate ethical approvals. This study followed the Strengthening of the Reporting of Observational Studies in Epidemiology (STROBE) reporting guidelines.

### Murine models and tissue processing

Human TNBC cell line MDA-MB-231 was purchased from the American Type Culture Collection (ATCC) and cultured in RPMI 1640 medium supplemented with 10% fetal bovine serum (FBS) and 1% penicillin/streptomycin antibiotic at 37°C and 5% CO_2_. TNBC cells (1 × 10^6^) were injected into the right flank of nude female mice, generating cancer cell line-derived xenografts referred to as the XEN models. Tumor growth was monitored weekly by measuring its width (W) and length (L). Tumor volume, V, was estimated using the formula V = π/6 × L × W^2^. Mice were sacrificed 45 days after injection. After euthanasia, primary tumors, lymph nodes, and lung metastases were collected. Samples were fixed in formalin, embedded in paraffin, and sectioned at four μm for histological analysis using H&E staining. Metastatic lesions were evaluated by a trained pathologist.

### Hematoxylin and eosin staining

Four μm sections were stained to identify cancerous sections in primary tumors, lymph nodes, and lung tissues. Slides were first deparaffinized using Clearify^™^ (American MasterTech #CACLELT, alternative to xylene; three times, five minutes each) and rehydrated through a graded ethanol series (100% three times; 95% once, 70% once, one minute each) followed by deionized H_2_O. Sections were stained with hematoxylin (VWR #95057–844) followed by rinsing in running tap water continuously for two minutes. Slides were dipped in differentiating solution (0.25% HCl in 70% ethanol) eight times. Again, slides were washed in tap water for one minute. Bluing was performed in alkaline water (1g/L of sodium bicarbonate in distilled water), placing the slides for 30 seconds. After that, the slides were washed in running tap water for three minutes. Slides were then placed in 70% ethanol for one minute with agitation. Then, samples were counterstained with eosin (VWR #95057–848) for one minute. Following eosin staining, sections were dehydrated again (four times in 100% ethanol, two minutes each) and dried on the bench overnight. The next day, samples were immersed in Clearify^™^ (twice, five minutes each) and mounted with coverslips using a permanent mounting medium (American MasterTech #MMC0226). Stained sections were examined using a brightfield microscope.

### DNA purification

Genomic DNA was extracted from eight μm tissue sections. Tumor and metastatic regions were microdissected with a needle, avoiding mouse tissue contamination, taking the labeled sections of H&E as reference. DNA was isolated using Quick-DNA FFPE Kit (ZYMO Research Cat. No: D3067) according to the manufactureŕs instructions. DNA concentration and purity were determined by Qubit 3.0 (Q33216; Thermo Fisher Scientific, CA) fluorometric quantification.

### Genome-wide DNA methylation profiling and data processing

DNAm patterns from our XEN models were assessed using the Infinium Methylation EPIC v2.0 array. Bisulfite conversion, amplification, and hybridization on the beadchip were performed by Diagenode Inc. following the manufacturer’s protocol.

Data import, quality control, and normalization were performed using the R package *ChAMP* (v2.36.0)^26^. Briefly, raw intensity data were imported and further processed using default settings. Beta-mixture quantile normalization was applied, and batch effects were tested and corrected using the “champ.runCombat” function. Probes with a detection P-value above 0.01 were excluded. Probes targeting non-CG dinucleotides, single-nucleotide polymorphisms, and chromosome Y were also removed, with a total of 868,873 CpG sites remaining for downstream analyses. β-values represent the ratio of the intensity of the methylated (M) bead types to the total intensity of both methylated and unmethylated (U) bead types at each CpG site. They can be calculated using the equation β = [M / (M + U)].

### Public data access and processing

AURORA US datasets, including DNAm data (Illumina HumanMethylationEPIC array) and transcriptome profiling (RNA-sequencing) from metastatic and primary BC tumors, were obtained from the NCBI-Gene Expression Omnibus (GEO; GSE212370 and GSE209998, respectively)^11^. Only samples defined as TNBC were included in this study (n = 39), consisting of 6 pre-treated primary tumors and 33 metastases (**Supplementary Table 1**).

DNAm data from the AURORA US cohort were processed as previously described. RNA-sequencing data were processed using the *DESeq2* (v1.42.1) R package^27^. Genes with a base mean of 20 reads or fewer were discarded.

Additional gene expression data (Affymetrix Human Genome U133A Array) from two independent TNBC cohorts, GSE2603 (n = 30)^15^ and GSE5327 (n = 58)^28^, were obtained from the NCBI-GEO. Briefly, raw CEL files were downloaded and imported into R using the *oligo* (v1.66.0) package. Data were normalized using the robust multi-array average method and subsequently merged, correcting for potential batch effects using the “ComBat” function from the *sva (v3.50.0)* R package^29^. Additionally, TNBC xenograft-derived data from the same GEO series (GSE2603)^15^ were retrieved and processed as previously described. This dataset includes parental MDA-MB-231 cell lines and derivatives isolated from lung-metastatic lesions. We included the parental cell lines (n = 2) and the LM0 and LM2 derivatives (n = 4) for the analyses.

Chromatin states for mammary epithelial cells were obtained from the Broad ChromHMM Track (hg19) on the UCSC Genome Browser^30^ and processed using the LiftOver tool in the Galaxy platform into hg38^31^. Subsequently, the data were summarized into biologically relevant states: promoters, enhancers, insulators, heterochromatin, and active transcription regions. The intersection between chromatin states and DNAm data for each CpG site was performed using the *GenomicRanges* (v1.54.1)R package^32^.

### Selection of relevant genes regulated by DNA methylation mechanisms

CpG genomic positions were explored in each probe using the improved DNAm Illumina EPIC v2.0 probe annotation^33^. To ensure robustness, we analyzed transcript isoforms for each gene, including only protein-coding transcripts. Promoter regions were defined as the 2,000 base pairs upstream of each transcription start site. The number of differentially methylated sites (DMS; absolute methylation difference > 15% and P-value < 0.05) per gene was then computed based on this criterion, excluding duplicated CpG sites. Genes with at least two DMS in their promoter regions were selected. This filtering step was applied to both our XEN model and the AURORA US dataset to identify protein-coding genes with recurrent epigenetic aberrations. Genes showing discordant methylation patterns (e.g., hypomethylated in XEN and hypermethylated in AURORA US) were removed from downstream analyses. Subsequently, we intersected this set with genes showing concordant changes in expression, specifically those exhibiting promoter hypomethylation coupled with upregulation, or hypermethylation coupled with downregulation. Visualization was restricted to promoter-associated CpG sites that were present on both EPICv1 and EPICv2 arrays to allow direct comparison.

### Functional enrichment analyses

Pathway enrichment analyses were conducted using the *clusterProfiler* (v4.8.3) R package^34^. The curated Cancer Hallmark dataset was obtained from the online tool www.cancerhallmarks.com^35^. Pathways included in the Kyoto Encyclopedia of Genes and Genomes (KEGG) dataset were sourced from the MSigDB database using the *msigdbr* (v. 7.5.1) R package^36^.

### Survival analyses

Lung metastasis-free survival (LMFS) was assessed using annotated clinical data from the GSE2603 and GSE5327 datasets (**Supplementary Table 2**). Survival differences between high and low gene expression groups were evaluated using log-rank tests. Additionally, Cox proportional hazards modeling was conducted to calculate hazard ratios (HR) between groups. For HR calculation, the low expression group served as the reference group. The online tool https://kmplot.com/analysis/ was used to perform Kaplan-Meier curves^37^. LMFS was defined as the length of time from diagnosis until the occurrence of lung metastasis or death from any cause. Because no events occurred beyond 60 months, the follow-up was restricted to 60 months.

### Data processing and visualization

Heatmaps were generated to display hierarchical clustering based on Euclidean distance using the R package pheatmap (v1.0.12). For visualization, the mean methylation of each CpG for each animal was used to normalize the data. Phylogenetic trees with Spearman ρ distances between DNAm profiles were generated using the FigTree software version 1.4.4 (Andrew Rambaut Lab, University of Edinburgh). Forest plots were generated using the forestploter (v1.1.2) R package. For data representation, raw counts were normalized using DESeq2 size factors, accounting for differences in library depth, and then log₂-transformed using a pseudocount of 1. For data representation, the *ggplot2* (v3.4.4)^38^, *circlize* (v0.4.16)^39^, *ggven* (v0.1.16), *ggpubr* (v0.6.0), *UpSetR* (v1.4.0)^40^, and *patchwork* (v1.3.0) R packages were used. The collection of R packages grouped on *tidyverse* (v2.0.0) was used for data management^41^.

### Statistics and reproducibility

DMS were identified using the two-sided Wilcoxon rank-sum test. The average β-value of each CpG site was compared between groups. For the initial comparisons between XEN carcinomas, CpG sites were considered DMS if they showed an absolute DNAm difference greater than 15% with a P-value below 0.1. For the joint comparisons and the AURORA US dataset, significance was defined as an absolute methylation difference greater than 15% with a P-value of less than 0.05. The chromosomal distribution of DMS was assessed by comparing observed versus expected DMS based on EPIC probe density. Per-chromosome enrichment was further tested using Fisher’s exact test. The odds ratios with 95% confidence intervals were calculated using the R package *questionr* (v0.7.8), with derived P-values obtained using Fisher’s exact test. For bulk RNA-sequencing data, differentially expressed genes were defined as those with an absolute log2 fold change (FC) > 1 and a Wald test P-value < 0.05. When necessary, P-values were corrected for multiple tests with the Benjamini–Hochberg procedure to calculate the false discovery rate. Unless otherwise specified, all computational analyses were performed using R software (v4.3.3), using a set of random seeds to ensure reproducibility. Key analyses were independently re-run during the course of the study to confirm consistent results.

## Supplementary Material

Supplementary Files

This is a list of supplementary files associated with this preprint. Click to download.

- BedoyaLopezetal.SupplementaryTable1.xlsx
- BedoyaLopezetal.SupplementaryTable2.xlsx
- BedoyaLopezetal.SupplementaryFigures.pdf

## Data Availability

Validation datasets used in this study are publicly available from the NCBI Gene Expression Omnibus (GEO) database. These include the AURORA US cohort (accession numbers GSE212370 and GSE209998) and TNBC primary tumor datasets with clinical annotations on lung metastasis events (GSE2603 and GSE5723). All datasets can be accessed at: https://www.ncbi.nlm.nih.gov/geo/. The newly generated epigenetic data from this study have been uploaded to the NCBI-GEO database under the private accession code GSE305869. In the interim, researchers seeking access to this data may submit a formal request to Diego M. Marzese, diego.marzese@idisba.es. Once the data is publicly available, it will be accessible via NCBI-GEO following the repository’s policy without restrictions.

## Code availability:

No specific code was generated for this project. All code used for R scripts was adapted from the standard scripts used in the packages, which are mentioned in the Methods section.
